# Effects of Badminton Expertise on Representational Momentum: A Combination of Cross-Sectional and Longitudinal Studies

**DOI:** 10.3389/fpsyg.2017.01526

**Published:** 2017-09-19

**Authors:** Hua Jin, Pin Wang, Zhuo Fang, Xin Di, Zhuo’er Ye, Guiping Xu, Huiyan Lin, Yongmin Cheng, Yongjie Li, Yong Xu, Hengyi Rao

**Affiliations:** ^1^Key Research Base of Humanities and Social Sciences of the Ministry of Education, Center of Cooperative Innovation for Assessment and Promotion of National Mental Health, Academy of Psychology and Behavior, Tianjin Normal University Tianjin, China; ^2^Guangdong Vocational College of Environmental Protection Engineering Foshan, China; ^3^Laboratory of Applied Brain and Cognitive Sciences, Shanghai International Studies University Shanghai, China; ^4^Center for Functional Neuroimaging, Department of Neurology, University of Pennsylvania, Philadelphia PA, United States; ^5^Department of Biomedical Engineering, New Jersey Institute of Technology, Newark NJ, United States; ^6^School of Psychology, South China Normal University Guangzhou, China; ^7^School of Education, Guangdong University of Education Guangzhou, China; ^8^College of Chinese Language and Culture, Jinan University Guangzhou, China; ^9^Institute of Applied Psychology, Guangdong University of Finance Guangzhou, China; ^10^National Badminton Team of China Beijing, China

**Keywords:** representational momentum, badminton training, causal relationships, transfer, cross-sectional, longitudinal

## Abstract

Representational momentum (RM) has been found to be magnified in experts (e.g., sport players) with respect to both real and implied motion in expert-familiar domains. However, it remains unclear whether similar effects can be achieved in expert-unfamiliar domains, especially within the context of implied motion. To answer this question, we conducted two independent experiments using an implied motion paradigm and examined the expert effects of badminton training on RM in both adult and child players. In Experiment 1, we used a cross-sectional design and compared RM between adult professional badminton players and matched controls. The results revealed significantly enhanced RM for adult players, supporting the expert effect in expert-unfamiliar domains for implied motion. However, cross-sectional studies could not ascertain whether the observed expert effect was due to innate factors or expertise acquirement. Therefore, in Experiment 2, we used a longitudinal design and compared RM between two groups of child participants, naming child players who had enrolled professional badminton training program at a sports school and age-matched peer non-players who attended an ordinary primary school without sports training. Before training, there were no differences in RM among child players, their non-player peers, and adult non-players. However, after 4 years of badminton training, child players demonstrated significantly enhanced RM compared to themselves prior to training. The increased RM observed in both adult and child players suggests that badminton expertise modulates implied motion RM.

## Introduction

When an object is moving from one side to the other, individuals often report the location of the object to be a bit further along in its trajectory. This physical momentum-like phenomenon in mental representation is referred to representational momentum (RM; Freyd, 1983, 1984; Freyd and Finke, 1984; Hubbard and Bharucha, 1988). Previous studies have consistently shown that stimulus characteristics and environment factors significantly affect RM (Hubbard, 1995, 2005, 2014), indicating a bottom-up processing of RM. Meanwhile, several studies, particularly with respect to fast ball sports, have found effects of observer expertise, suggesting a top-down processing of RM (e.g., Tresilian, 1995; DeLucia and Liddell, 1998; Didierjean and Marmèche, 2005; Nijhawan, 2008; Nijhawan and Wu, 2009; Blättler et al., 2010, 2011, 2012; Gorman et al., 2011, 2012; Nakamoto et al., 2015). When interpreting the effects of experts on RM processing, one assumption is that players must make appropriate cognitive extrapolation to anticipate the location of a moving ball and interact optimally with a fast-moving ball (Tresilian, 1995; DeLucia and Liddell, 1998; Hubbard, 2005; Nijhawan, 2008; Nijhawan and Wu, 2009; Nakamoto et al., 2015). Consequently, the memory of the final position of a moving ball may be displaced further along the path of motion (i.e., the RM may be larger) for the players compared to the controls. Consistent with this assumption, in a study by Didierjean and Marmèche (2005), participants were presented with two sequential configurations about basketball matches and asked to judge whether the second configuration was the same as the first one. When the second configuration was the next-likely state of the first configuration, experienced basketball players responded less accurately and more slowly compared to novices. In addition, experts more frequently failed to recognize new configurations when these configurations were the next-likely state of an already encoded configuration. Similarly, when Gorman et al. (2012) asked expert and novice basketball players to view movie clips about static and moving patterns with regards to basketball and then to recall the positions of the actors, experts reported the locations of the actors significantly further in advance of the actual location than did novices. Additionally, Blättler et al. (2010, 2011, 2012) reported that experienced drivers and pilots exhibited enhanced RM in their familiar scenes (e.g., a landing aircraft) than did novices. These findings support that RM is enhanced by sport expertise, particularly within expert-familiar domains.

Previous studies have investigated the expert-related RM effects in unfamiliar domains as well, though the findings remain controversial. For example, Gorman et al. (2011) found that football players exhibited no RM effect in the basketball-related task, whereas baseball experts exhibited enhanced RM (cognitive extrapolation) than did novices when judging positions of a moving target (Nakamoto et al., 2015). The discrepant findings may be relevant to whether experts have superior ability with respect to fast action anticipation. For example, in fast ball (e.g., baseball) and close combat sports (e.g., karate), players should possess a strong ability to anticipate fast actions, as they must process the moving information quickly to successfully and appropriately react. This assumption is consistent with a study by Rosalie and Müller (2014), which found that only karate experts but not near-experts could perform like domain experts in the Australian football transfer domain.

Most previous studies examined the expert-unfamiliar RM effects with respect to real motion. To the best of our knowledge, only one study (Blättler et al., 2010) examined the RM effects related to implied motion. In this study, experienced and inexperienced drivers were presented with video clips consisting of a running person or a moving geometric object and a test image after each clip. The test image was either identical or forward- or backward-shifted to the last image of the clip. This study failed to find any differences in RM between the experienced and inexperienced drivers.

The ability of anticipating fast actions plays an important role in the effects of sport expertise on RM. Hence, the absence of RM effects in Blättler et al. (2010) study may be attributed to the poor ability of drivers to anticipate action. Therefore, the first aim of the present study was to further investigate whether experts compared to novices would exhibit enhanced RM in expert-unfamiliar domains for implied motion by recruiting experts who exhibited superior ability in the area of fast action anticipation. Our previous study on action anticipation (Jin et al., 2011) compared professional badminton players to non-players and revealed that players have greater ability to anticipate fast actions. In Experiment 1 of this study, we used a cross-sectional design and compared the RM between adult professional badminton players and non-players. Based on previous findings (e.g., Didierjean and Marmèche, 2005; Blättler et al., 2010, 2011, 2012; Gorman et al., 2012; Nakamoto et al., 2015), we predicted that experienced badminton players would exhibit a significantly greater RM magnitude than non-players. However, cross-sectional studies could not exclude the influence of innate factors. To resolve this issue, Experiment 2 used a longitudinal design and examined the changes in RM among a cohort of child players before and after 4 years of badminton training, i.e., in the first and the second measurements, respectively, and explored the difference in RM between child players and peer non-players. We expected that there would be no difference in RM between child players and peer non-players in the first measurement, but the RM would be significantly greater for child players in the second measurement compared to the first.

## Experiment 1

### Methods

#### Participants

Twenty adult professional badminton players (10 males, *M* ±*SD* = 22.48 ± 4.57 years) were recruited from provincial/municipal badminton teams in China. All players had at least 5 years’ of professional training and were qualified as a national player (in second grade or above). In the last 2 years, they participated in at least three 2-h training sessions per week. The non-player control group included 19 undergraduate students (9 males, 20.90 ± 4.19 years), none of whom had received any professional training in any ball sports. The two groups were matched in age and education (both *p* > 0.2). All participants were right-handed, had normal or corrected-to-normal vision, and reported no history of neurological illness. All participants gave informed consent prior to the experiment. This study was approved by the Ethics Committee of the School of Psychology, South China Normal University. The entire experimental protocol was conducted according to the approved guidelines, which were in accordance with the Declaration of Helsinki.

#### Experimental Procedure

Prior to the formal experiment, participants were asked to complete a questionnaire regarding their experiences in sports and then complete 20 practice trials. The participants were individually assessed in a dimly illuminated, sound-attenuated room. Stimuli were presented using E-prime 2.0 software (Psychology Software Tools, Inc., Pittsburgh, PA, United States) on a black screen in the center of a monitor with a screen resolution of 1024 pixels × 768 pixels. Viewing distance was approximately 100 cm.

**FIGURE 1:**
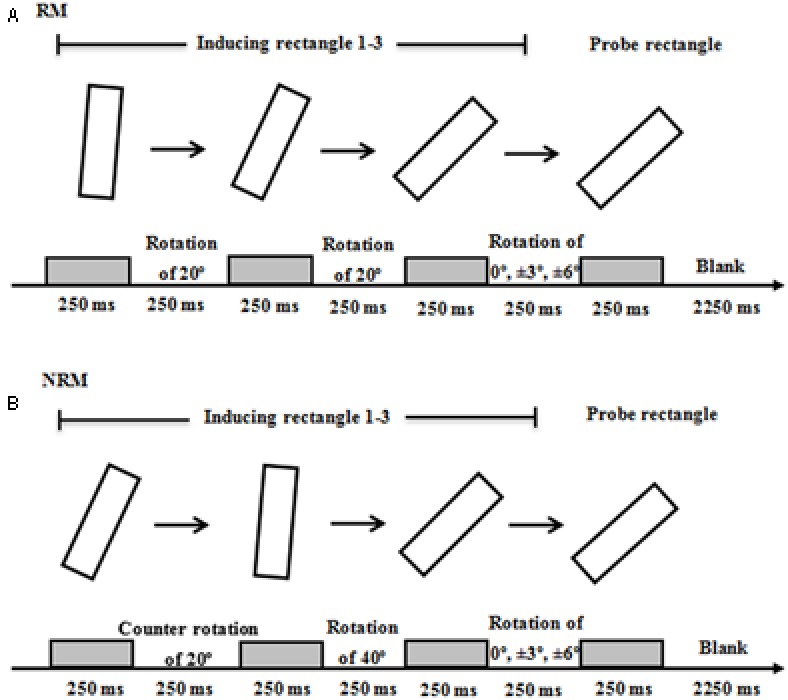
Schematic illustration of the display sequence used in two experiments. **(A)** RM and **(B)** NRM were examples of a display sequence for RM and NRM conditions, respectively. The three rectangles plus the probe rectangle, presented sequentially in the experiments, were displayed from left to right. The probe on the far right, in its orientation, matched that of the third inducing rectangle (i.e., the angular distance between the third inducing rectangle and the probe was 0°). Participants were instructed to view the four presentations and then to judge, as quickly as possible, whether the probe had the same orientation as the third inducing one. The next trial was initiated either after their response or after a 2250 ms presentation of a blank screen following the probe.

In the present study, we used an implied-motion RM task paradigm adopted from a previous study (Freyd and Finke, 1984). **Figure 1** illustrates the time course of the stimulus presentation for each condition. As presented in **Figure 1**, three inducing rectangles at different orientations were presented successively to produce a consistent “implied rotation.” For the RM task, the inducing rectangles were oriented at 5°, 25° and 45°, successively, from the vertical (0°). Subsequently, a probe rectangle was presented. The probe rectangle was slightly forward or backward relative to the implied rotation. The orientation difference between the target and the third probe could be -6°, -3°, 0°, 3°, 6°. Each rectangle was presented for 250 ms and the time intervals between the presentation of two successive rectangles was also 250 ms. A blank screen was presented for 2250 ms before the onset of the next trial. During the presentation of the blank screen, participants were asked to indicate whether the probe was in the same orientation as the third inducing rectangle. The experiment included three sessions. In each session, there were three RM blocks, each of which consisted of 15 trials. A fixation point was presented in the center of the screen for 2 s prior to each block. The experiment took approximately 10 min.

#### Statistical Analysis

The RM magnitudes (in degrees) were quantified for each individual by calculating the weighted mean estimates of the memory shift, i.e., the sum of the products of the proportion of the same responses and the distance of the probe from true-same, in degrees, divided by the sum of the proportions of the same responses (Rao et al., 2004). If the inducing rectangles implied a consistent rotation in the RM task, the weighted mean estimates of the memory shift would be greater than zero.

For each group, one sample *t*-test was used to analyze whether the value of RM was different from zero, in order to determine whether experts and novices produced RM. To understand whether the magnitude of RM was different for experts compared to novices, the value of RM was entered into an independent-samples *t-*test (two-tailed) with group (players versus non-players) as the between-subject factor. Correlation analyses were also conducted to examine the relationships between badminton training duration and the magnitude of RM in the player group.

### Results and Discussion

**FIGURE 2:**
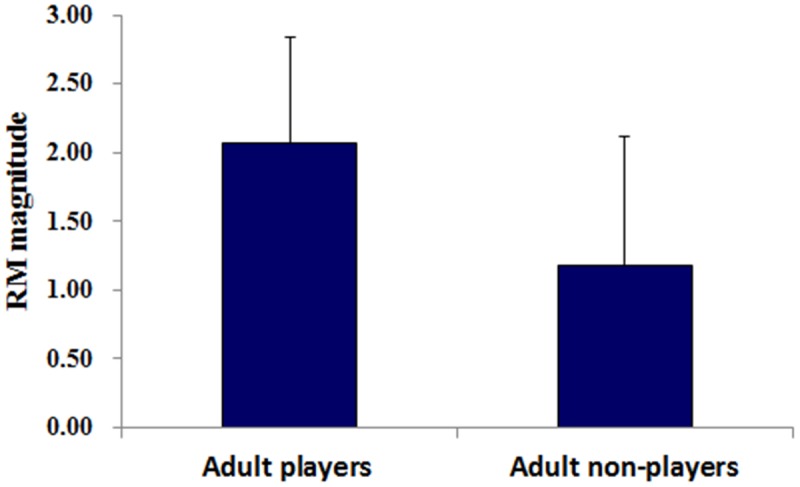
The mean of RM for adult badminton players and non-players. Vertical lines indicate the standard deviation of the mean.

The mean values (standard deviation, SD) of RM for adult non-players and players were 2.07 (0.77) and 1.18 (0.93), respectively (**Figure 2**). One sample *t*-tests indicated that these RM values were significantly higher than zero for both groups [for non-players: *t*(18) = 5.54, *p* < 0.001, *d* = 1.27; for players: *t*(19) = 12.01, *p* < 0.001, *d* = 2.69], indicating that both badminton players and novices exhibited RM. Comparison between two groups showed a greater RM magnitude for players than non-players [*t*(37) = 3.25, *p* = 0.002, *d* = 1.05], suggesting that RM magnitude is enlarged by badminton expertise. Therefore, the findings suggest that the expert effects of RM can be transferred to expert-unfamiliar domains within the context of implied motion. However, the correlation analyses did not found significant correlations between badminton training duration (including professional training duration and amateur training duration) and the magnitude of RM (*p* > 0.05).

Although we found significantly enlarged RM for adult professional badminton players than adult non-players in Experiment 1, the cross-section design used here does not allow for the exclusion of influencing innate factors. For example, the experts may be born with superior abilities related to RM. Therefore, in Experiment 2, we employed a longitudinal design, in which individuals were measured in RM before and after acquisition of expertise regarding RM, to rule out the potential influences of innate factors.

## Experiment 2

### Methods

#### Participants

Seventeen child players (8 males; 10.82 ± 0.73 years) and 32 peer non-players (18 males; 11.03 ± 0.57 years) were pre-selected based on their responses to a sports experience questionnaire. Players and peer non-players were similar in age and education (both *p* > 0.2). The child players met the following inclusion criteria: (1) currently attended a local sport school; (2) participated in an amateur badminton training program for less than 10 months; (3) practiced at least three times per week and 2 h per practice for the following 4 years after first test. The peer non-players had no professional training experience in any ball sports and were recruited from an ordinary primary school. Both players and non-players participated in the first measurement. The second assessment was scheduled to occur 4 years later. Unfortunately, we failed to obtain the data from the non-players for the second measurement when they entered different junior high schools. In addition, five of the players quitted the training program before the time of the second assessment. Hence, we were able to gather data from only 12 of the original players (6 males; 14.42 ± 0.79 years). All participants gave informed consent prior to each measurement in the presence of their guardians (i.e., their teachers or coaches). This study was approved by the Ethics Committee of the School of Psychology, South China Normal University. The experiment was conducted in accordance with the approved guidelines and protocols of the Declaration of Helsinki.

#### Experimental Procedure

The procedure was the same as that in Experiment 1.

#### Statistical Analysis

The RM values were calculated for each participant and each measurement, and one sample *t*-tests were first conducted to analyze the differences between the RM values and zero to ascertain whether child players and non-players produced RM. Paired *t-*test with measurement (first versus second) as within-subject factor was then used to examine whether professional badminton training increased the value of the RM in child players. A one-way ANOVA was conducted to compare the RM values between child players, child non-players, and adult non-players (from Experiment 1), in order to determine whether RM differed between these groups even before training (i.e., affected by innate factors). Independent-samples *t-*test was also used to compare the RM between adult non-players and child players after training.

### Results and Discussion

**FIGURE 3:**
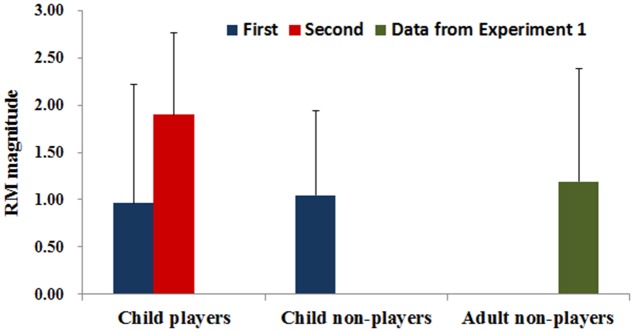
The mean of RM for child players, child non-players and adult non-players. Vertical lines indicate the standard deviation of the mean.

For the first measurement, the mean values (SD) of RM for child players and child non-players was 1.19 (1.19) and 1.05 (0.89), respectively. For the second measurement of child players, the mean RM value (SD) was 1.90 (0.87) (**Figure 3**). All these RM values were significantly larger than zero [non-players: *t*(31) = 6.63, *p* < 0.001, *d* = 1.18; players in the first measurement: *t*(16) = 4.12, *p* < 0.005, *d* = 1.00; players in the second measurement: *t*(11) = 7.57, *p* < 0.001, *d* = 2.18], indicating that RM was produced for children non-players and players before and after 4 years of professional training.

Paired *t*-test comparison showed that the RM magnitude was larger for child players in the second measurement compared to the first measurement [*t*(11) = 3.22, *p* = 0.008, *d* = 0.87], suggesting that long-term professional badminton training did enhance the RM for badminton players. However, the ANOVA analysis revealed no difference [*F*(2,67) = 0.17, *p* = 0.849] among RM in the three groups (child players in the first measurement, child non-players, and adult non-players), indicating that both child players and non-players reached the level of adult non-players in RM.

The analysis from the independent-samples *t*-test indicated that child players measured in the second measurement exhibited greater RM than did adult non-players [*t*(29) = 2.13, *p* = 0.042, *d* = 0.79]. These findings suggest that long-term professional badminton training enhances the RM of badminton child players to a point where it surpasses that of adult non-players.

## General Discussion

The present study investigated the effects of badminton expertise on RM. Using a cross-section design in Experiment 1, we found a larger RM magnitude for adult badminton players compared to non-players. Using a longitudinal design in Experiment 2, we found that child players produced enhanced RM after 4 years of professional badminton training, and the magnitude of RM was greater than that of adult non-players. However, there were no differences in RM for the child players before training when compared to peer non-players and adult non-players. These findings provide consistent evidence supporting that the modulation of expertise on RM with regards to implied motion can be transferred into expert-unfamiliar domains.

Why does badminton expertise enlarge RM magnitude in the implied motion RM task? One possible reason is that players develop enhanced motor skills and sensitivity to motion information as a result of their years of sport training. Developmental studies have revealed that the RM is modulated by motor skills. For example, individuals who have defects in motion processing, such as preterm children, (Taylor and Jakobson, 2010) and children with dyslexia (Barnes et al., 2007) or developmental disabilities (Conners et al., 1997), exhibited reduced RM. Consequently, badminton players have better motor skills compared to non-players, thus they may exhibit greater RM than non-players.

While sport players should possess superior motor skills, action anticipation may be one of the most important skills that influences RM. Fast ball sports (e.g., badminton and basketball) and close combat sports (e.g., karate) are characterized by severe time constraints on intercepting a moving object. Due to neural delay processing in the visuo-motor system (Khurana and Nijhawan, 1995; Nijhawan, 2008; Nijhawan and Wu, 2009; Zago et al., 2009), players must anticipate the outcome of the opponent’s sequential movement (e.g., the moving trajectory of an object) at the right point to react successfully. Therefore, players should acquire superior ability of action anticipation (Goulet et al., 1989; Abernethy, 1990a,b; Williams et al., 1994; Ripoll et al., 1995; Singer et al., 1996; Williams and Davids, 1998; Williams and Elliott, 1999; Abernethy et al., 2001; Ward et al., 2002; Laurent et al., 2006; Shim et al., 2006). This anticipation usually precedes the real moving trajectory, which may play an important role in extrapolating individual’s memory for the location of moving targets that lead to RM. Consistent with this hypothesis, previous studies have found that experienced players compared to non-players predicted an object’s final landing position much more accurately (Jin et al., 2010, 2011), suggesting that experts in fast ball sports may have increased ability to anticipate about the future location of a fast-moving object. In the present study, professional players may also be superior in the ability to anticipate an action, resulting in an enlarged magnitude of RM.

Our findings are in contrast to the studies of Blättler et al. (2010) and Gorman et al. (2011), which suggest that such expert effects of RM were experience-dependent and could not transfer to domains outside the expert’s area of expertise. However, findings from two other studies suggested the transferable expert effect of RM (Rosalie and Müller, 2014; Nakamoto et al., 2015). As mentioned before, recruiting experts who are superior in action anticipation may be an important factor for a transferable expert RM effect. Experienced drivers in Blättler et al. (2010) study might not be superior in this ability, thus they could fail to exhibit a transferable expert effect on RM. In the present study, however, professional badminton players had superior ability in fast action anticipation (Jin et al., 2011). Therefore, it is not surprising that we observed the expert RM effects in expert-unfamiliar domains for implied motion.

Moreover, while previous studies and Experiment 1 in the present study demonstrated enhanced RM for experts compared to novices, all of the studies used the cross-sectional design, in which the expert effects were investigated by observing the RM of experts compared to that of novices. However, it remains unclear whether the expert RM effect is due to expertise acquirement or innate factors (e.g., players may be born with increased RM). To rule out this possibility, Experiment 2 directly investigated whether expertise acquirement (e.g., training) modulated RM. The findings indicated enhanced RM for child players following extended professional training that even over-passed the RM of adult non-player. More importantly, the RM of child non-players was found to be similar to that of child players before these players underwent long-term training. Taking together, these findings suggest that the innate factors cannot account for the observed expert RM effect.

Although our findings suggested that the expert RM effect in expert-unfamiliar domains was relevant to sport expertise, it remains unclear whether this effect is influenced by the similarity of different areas of expertise and the professional level of experts. Rosalie and Müller (2012, 2014) found that karate experts and near-experts were able to use visual information to anticipate and guide motor skill responses as domain experts, but only karate experts could perform similarly to domain experts in the football transfer domain. These findings indicate that the similarity of expertise between expert-familiar and expert-unfamiliar domains and the professional level influence the ability to anticipate action, which, as a result, may alter the RM effect. Future studies may manipulate these two factors to further investigate the related issues.

There are several limitations in this study. The first limitation is that we only measured RM in non-player peers once. Larger RM effects have been reported for 8-year-old children than for 5.5-year-old children (Taylor and Jakobson, 2010), suggesting that the RM may be in development in childhood. Because we were not able to measure the RM changes in this group after 4 years without training, we cannot rule out the possibility that the observed increases in RM in the child players after 4 years of badminton training may be due to the change in age. However, previous studies have indicated that RM did not differ between 8-year-old children and adults, suggesting that RM may already reach a plateau after the age of 8 (Futterweit and Beilin, 1994). In addition, we found no differences among child players, child non-players, and adult non-players, suggesting that the age effect cannot account for the observed training induced RM enhancements in this study. Second, the sample size is relatively small, especially for the group of child players whose RM was measured both before and after training. Therefore, future studies with larger sample sizes are needed to replicate findisng from the present study. Finally, badminton is only one type of sport expertise. As previously mentioned, the RM effects of sport training may be due to the enhanced ability of anticipation during the training. However, this ability is not enhanced by all sports, such as running. Further examination of RM effects in individuals who are trained in those sports may provide a better understanding of the important role of action anticipation ability in RM.

## Conclusion

The present study found that adult badminton players as compared to non-players produced enhanced RM with regards to implied motion. In addition, we also observed that RM was enhanced for child badminton players after 4 years of training, surpassing that of adult non-players. However, before training, there was no RM difference between child players and their non-player peer. Taken together, these findings indicate that badminton expertise may modulate RM in expert-unfamiliar domains.

## Author Contributions

HJ and HR were involved in study design, execution and manuscript drafting and revising. PW, ZF, XD, ZY, and GX were involved in execution, data analysis and manuscript revising. HL, YC, YL, and YX were involved in manuscript drafting and revising. We have read and approved the manuscript and agree to be accountable for all aspects of the work in ensuring that questions related to the accuracy or integrity of any part of the work are appropriately investigated and resolved.

## Conflict of Interest Statement

The authors declare that the research was conducted in the absence of any commercial or financial relationships that could be construed as a potential conflict of interest.
